# Respiratory infections and pneumonia: potential benefits of switching from smoking to vaping

**DOI:** 10.1186/s41479-016-0001-2

**Published:** 2016-04-12

**Authors:** Davide Campagna, Maria Domenica Amaradio, Mark F. Sands, Riccardo Polosa

**Affiliations:** 1 Dipartimento di Medicina Clinica e Sperimentale, Università di Catania, Catania, Italy; 2 UOC di Medicina Interna e d’Urgenza, Edificio 4, Piano 3, Azienda Ospedaliero-Universitaria “Policlinico-V. Emanuele”, Catania, Italy; 3 Jacobs School of Medicine and Biomedical Sciences, State University of New York, Buffalo, NY USA; 4 The Veterans Administration Healthcare System of Western New York, Buffalo, NY USA

**Keywords:** Respiratory infection, Pneumonia, Cigarette smoking, Electronic cigarette, Tobacco harm reduction, e-vapour

## Abstract

Abstaining from tobacco smoking is likely to lower the risk of respiratory infections and pneumonia. Unfortunately, quitting smoking is not easy. Electronic cigarettes (ECs) are emerging as an attractive long-term alternative nicotine source to conventional cigarettes and are being adopted by smokers who wish to reduce or quit cigarette consumption. Also, given that the propylene glycol in EC aerosols is a potent bactericidal agent, switching from smoking to regular vaping is likely to produce additional lung health benefits. Here, we critically address some of the concerns arising from regular EC use in relation to lung health, including respiratory infections and pneumonia. In conclusion, smokers who quit by switching to regular ECs use can reduce risk and reverse harm from tobacco smoking. Innovation in the e-vapour category is likely not only to further minimise residual health risks, but also to maximise health benefits.

Cigarette smoking is a known risk factor for pneumonia, with the risk increasing according to the intensity of smoking [1, 2]. A dose–response effect of cigarette smoking upon pneumonia risk has been reported in several studies, and summarised in a recent review [3]. The increased susceptibility to infection with a variety of bacterial and viral pathogens appears to be secondary to the harmful effect of tobacco smoke upon innate and adaptive immunity [4]. Undoubtedly, quitting smoking is among the most important steps smokers can take to lower the risk of respiratory infections and pneumonia. Indeed, ex-smokers have a lower risk of pneumonia than current smokers, irrespective of age [1, 2, 5]. Smoking is a difficult addiction to break with many smokers persisting in tobacco use for several years, and typically cycling through multiple periods of remission and relapse [6]. This is not surprising given the powerful addictive qualities of nicotine and non-nicotine sensory and behavioural cues [7]. Pharmacotherapy combined with behavioural smoking cessation support may double or triple quit rates [8], but efficacy rates, obtained in the context of rigorous randomised controlled trials, are not replicated in real-life, with disappointingly low cessation rates of about 4–5 % [9–11].

Electronic cigarettes (ECs) are consumer products consisting of a battery and a heating element (atomiser). Puffing on an EC heats up an element (the coil within the atomiser) that vapourises a solution (e-liquid) mainly consisting of propylene glycol, vegetable glycerin, distilled water, and flavourings that may or may not contain liquid nicotine. ECs are an attractive long-term alternative nicotine source to conventional cigarettes because of their many similarities with smoking behaviour [12, 13]. ECs come in a large variety of designs, shapes and sizes, some resembling conventional tobacco cigarettes (“cigalikes” ECs), others often resembling a pen (“penlike” ECs); however, most experienced users prefer more advanced designs that bear little visual resemblance to conventional cigarettes and allow customisation (e.g. larger-capacity batteries with adjustable power delivery, specific heating coils and multiple wick configurations). The large popularity of these products shows that smokers are now ready for this alternative form of smoking with the aim of reducing cigarette consumption, and to relieve tobacco withdrawal symptoms [12, 13]. Recent internet-based surveys [14, 15] and clinical trials [16, 17] confirm that ECs may help smokers to reach these goals.

In itself, abstaining from tobacco smoking by switching to ECs is likely to lower the risk of respiratory infections and pneumonia. Also, given that propylene glycol in its aerosol form is a potent bactericidal agent [18], regular vaping may have additional theoretical health benefits. Despite the good safety profile of propylene glycol, a hypersensitivity response to propylene glycol aerosols may occur in predisposed individuals. Propylene glycol is a common but often unrecognised cause of allergic contact dermatitis due to cosmetic products [19] and direct exposure to propylene glycol has been reported to cause signs and symptoms of irritation compatible with contact dermatitis around the mouth or in the oral mucosa of EC users [20]. Only one study (to date) models the interaction between EC aerosol emissions and susceptibility to airway infection. Sussan et al. [21] exposed mice to EC vapour for 2 weeks, followed by pneumococcal infection. EC-exposed animals had increased pneumococcal colony forming unit counts in both the airway and lung tissue. There are several problems with the design of the study and the authors’ interpretation of the results. The mice in the experimental group were exposed to a much higher level of stress than the control group, and stress affects bacterial and viral response. Moreover, the experimental conditions exposed the animals to significant nicotine poisoning, with an average cotinine concentration of 267 ng/ml. Cotinine is the primary metabolite of nicotine and in humans the amount of nicotine needed to give similar cotinine levels are tolerated by heavy smokers, but highly aversive to non-smokers, who would be expected to feel sick and vomit at this level of exposure. Mice are much more sensitive to nicotine than humans (lethal dose in mice is 3 mg/kg, in humans it is 6.5–13 mg/kg) [22]. The observed accelerated weight loss, reduced immunity and early death in the experimental group were more likely the result of protracted stress and nicotine poisoning.

These interesting findings in rodents do not corroborate the evidence in humans. Despite millions of regular EC users, there has been no evidence of new emerging pneumonia outbreaks in recent years, or reports of infectious pneumonia in the medical literature. However, we are aware of a case of lipoid pneumonia that respiratory physicians in the United States have suggested being a direct consequence of vaping [23]. Lipoid pneumonia is a rare respiratory illness that may occur from aspiration or inhalation of fatlike material in the lung; an occurrence that is commonly reported in elderly people after accidental ingestion of oil-based laxatives. The assertion that vaping could put people at risk of lipoid pneumonia is illogical, simply because commercially available e-liquids do not contain nor generate fatlike material. A more plausible cause for this patient’s lipoid pneumonia was the acute exposure to fumigation chemicals, which may contain crude essential oils.

Another area of concern is that of flavouring-induced lung disease. Food flavourings are safe to eat, but we don’t know whether that will also be true if they are inhaled. For example, diacetyl is a compound commonly used in the food industry that gives food a buttery taste. However, chronic exposure to high levels of this flavouring substance in microwave popcorn workers has been shown to cause the development of bronchiolitis obliterans, (“popcorn lung”) [24, 25]. Thus, it is of concern that many brands of vaping liquids may contain varying concentrations of diacetyl (particularly in nutty flavours) [26, 27]. Nonetheless, there is no report that this has caused bronchiolitis obliterans in EC users. It must be noted that cigarette smoke also contains diacetyl, but at a much higher level (up to 750 times higher) than those produced by ECs [28]. And yet there is no evidence that cigarette smoking causes bronchiolitis obliterans.

It has also been suggested that vaping may produce an aerosol of ultrafine particles, which could have negative effects on the lung health of bystanders [29]. However, it is not the size that is important, but the composition of the particle that matters; ultrafine particles are known to be generated in large quantities when boiling water [30], but water vapour particles are not harmful to the respiratory system. Particles emitted from ECs have completely different physical (they are liquid droplets) and chemical (they have hardly any of the toxic properties) properties compared to environmental pollution or cigarette smoke [31]. Most importantly, passive vaping cannot be compared to passive smoking, because ECs do not have a substantial negative impact on indoor air quality given that – by design – they do not generate side-stream emissions [32]. Finally, there are no studies indicating that particles emitted from ECs represent a risk factor for bystanders.

Considering that vapour toxicology is by far less problematic than that of conventional cigarettes [29], that e-vapour products are at least 96 % less harmful compared to combustible cigarettes [33] and that cigarette smoking carries a heightened risk for respiratory infections and pneumonia [1–4], substantially reducing daily cigarette consumption in smokers by switching to e-vapour products is likely to produce significant health benefits. Most importantly, any residual risks will be further diminished by adopting new technologies and applying quality and safety standards. ECs will be soon regulated by the new Tobacco Products Directive (TPD) of the European Parliament and the Council of the European Union [34], which mandates that e-vapour products are only placed on the market if the nicotine dose and uptake is reported on the unit packet and toxicological risk assessment is carried out on aerosol emissions.

Fast innovation in the e-vapour category is likely not only to further minimise residual health risks, but also to maximise health benefits in regular EC users. For example, by exploiting different product designs, we are now beginning to learn that adoption rates (and consequently the extent of reduction in tobacco consumption) are strongly associated with their efficiency as smoking cessation products, where smoking cessation is the main “collateral benefit” for many smokers switching to regular EC use [35–37]. Future research will have to quantify the extent of achievable reduction of the risk when switching to regular EC use. These research endeavors, together with the emerging positive evidence in EC users with preexisting airway diseases [38], may consolidate the notion that smokers who quit smoking by switching to ECs can reverse harm from tobacco smoking in the lung [39]. This should be taken into consideration by regulatory authorities seeking to adopt proportional measures for the e-vapour category [40].
